# Tannic acid delayed dietary fiber fermentation: dual mechanisms of enzyme inhibition and microbial community dynamics

**DOI:** 10.3389/fmicb.2025.1751028

**Published:** 2026-01-07

**Authors:** Liping Ren, Haoqiang Wang, Shunjing Luo, Chengmei Liu, Xiuting Hu

**Affiliations:** 1 State Key Laboratory of Food Science and Resources, Nanchang University, Nanchang, China; 2 International Institute of Food Innovation, Nanchang University, Nanchang, Jiangxi, China

**Keywords:** dietary fibers, glycoside hydrolases, gut microbiota, tannic acid, the fermentation rate

## Abstract

This study examined the influence of tannic acid (TA) on the fermentation rate of inulin (INU), fructooligosaccharides (FOS), galacto-oligosaccharides, xylo-oligosaccharides, isomalto-oligosaccharides, and *β*-glucan. It was found that 1.5 μmol/mL TA decreased production of gas and short-chain fatty acids from fermentation of these fibers, confirming that TA decreased the fermentation rate. Moreover, 1.5 μmol/mL TA inhibited the activity of *β*-fructosidase, xylanase, *β*-galactosidase, *α*-glucosidase, and *β*-glucanase secreted by gut bacteria and decreased the fermentation rate of glucose, fructose, xylose, and galactose. In summary, TA inhibited the activity of enzymes that participated in metabolism of dietary fibers and thus decreased the fermentation rate. On the other hand, INU and FOS were selected as representatives of dietary fibers, and the effect of interplay between TA and INU or FOS on the composition of gut microbiota was analyzed. TA and FOS or INU selectively promoted the growth of different specific genera, which might also result in the decrease in the fermentation rate. Moreover, co-fermentation of TA and FOS or INU increased the abundance of beneficial bacteria, including *Faecalibacterium* and *Coprococcus*, at the late fermentation stage, which suggest that decreasing the fermentation rate of fibers was beneficial for the distal colon health.

## 1 Introduction

Gut microbiota play a critical role in regulating human metabolism and health. Recent studies have demonstrated that dysbiosis of gut microbiota is strongly associated with chronic diseases such as inflammatory bowel disease, obesity, type 2 diabetes, and colorectal cancer (Mishra et al., 2023; Ni et al., 2017; Wong and Yu, 2023). Microbial imbalance may compromise intestinal barrier integrity and induce systemic inflammation, thus accelerating disease progression. Dietary fibers, a primary energy substrate for gut microbiota, are one of the most influential factors shaping the composition of gut microbiota. Fermentation of dietary fibers produces short-chain fatty acids (SCFAs), such as acetate, propionate, and butyrate. These metabolites supply energy to colonic epithelial cells and exhibit the anti-inflammatory activity, thereby preventing the above diseases (Goushki et al., 2025; Nogal et al., 2021).

Regardless of whether dietary fibers are water-soluble or not, their fermentable part is primarily metabolized in the proximal colon (van Deuren et al., 2025; Wang et al., 2019). This leads to insufficient supply of fermentable carbohydrates in the distal colon, and gut microbiota have to metabolize proteins as an energy source. Such proteolytic fermentation generates harmful metabolites, including ammonia, *p*-cresol, and branched-chain fatty acids, which increase the risk of colitis and colon cancer in the distal colon (Boven et al., 2025). However, high intake of dietary fibers may lead to excessive production of gas and accumulation of a large amount of SCFAs in a short time, which may exceed the threshold tolerance to the host and thereby aggravate colon inflammation (He X. et al., 2023; He X. X. et al., 2023; Wang et al., 2021). For instance, high intake of fructooligosaccharides not only failed to alleviate symptoms but also aggravated colonic damage in rats (Geier et al., 2007). Similarly, high intake of guar gum-containing diet led to immune overactivation, epithelial damage, and infection, thereby aggravating the inflammatory response of mice (Nair et al., 2021). Thus, it is necessary to slow down fermentation of dietary fibers and deliver dietary fibers to the distal colon.

The metabolic process of dietary fibers is divided into two stages. Dietary fibers are initially hydrolyzed by gut microbiota into monosaccharides. Subsequently, monosaccharides are further metabolized via glycolysis or the pentose phosphate pathway to generate SCFAs, etc. The whole metabolic process of dietary fibers is regulated by a series of enzymes. Therefore, it is possible to reduce the fermentation rate of dietary fibers by inhibiting the activity of these enzymes. Polyphenols are widely distributed in plant-based food. It has been widely reported that polyphenols inhibit the amylase activity in the upper digestive tract and thus delay starch digestion (Cao et al., 2023; Deng et al., 2023). Therefore, we inferred that polyphenols could inhibit the activity of amylase secreted by gut bacteria and thereby slow down fermentation of resistant starch, which was confirmed by *in vitro* and *in vivo* experiments (Liu et al., 2024a, 2024b). Similarly, we inferred that polyphenols could also inhibit the activity of other glycoside hydrolases and thus slow down fermentation of other dietary fibers.

On the other hand, only 5–10% of ingested polyphenols are absorbed in the small intestine, while 90–95% reach the colon for microbial metabolism (Gowd et al., 2019; Xie et al., 2023). Accumulating evidence from *in vitro*, animal, and human studies highlights the beneficial effects of polyphenols on the composition of gut bacteria (Lan et al., 2024; Liang et al., 2024; Zhou et al., 2025). Moreover, polyphenols and dietary fibers may simultaneously reach the colon (Phan et al., 2017; Rodríguez-Daza et al., 2020). It has also been reported that co-fermentation of dietary fibers and polyphenols is beneficial for the host health (Le et al., 2024; Loo et al., 2022), which may also be associated with the ability of polyphenols to slow down fermentation of dietary fibers. Tannic acid has been found in a variety of plants utilized as food (Chung et al., 1998). These include grains, fruit, wines and tea. Moreover, tannic acid is cheap. Therefore, this work aimed to investigate the impact of tannic acid, a representative of polyphenols, on the fermentation rate of other common dietary fibers.

## 2 Materials and methods

### 2.1 Materials

Fructooligosaccharides (FOS) (Orafti^®^ P95; degree of polymerization (DP) 2–8; purity ≈ 95%) and inulin (INU) (Orafti^®^HP; DP ≥ 23; purity ≈ 100%) were sourced from Beneo (Tienen, Belgium). Galacto-oligosaccharides (GOS) (DP 2–8; purity ≥ 70%), isomalto-oligosaccharides (IMO) (DP 5–11; purity ≈ 90%), and xylo-oligosaccharides (XOS) (DP 2–7; purity ≈ 95%) were obtained from Shanghai Yuanye Bio-Technology Co., Ltd. (Shanghai, China), and oat *β*-glucan (BG) (the molecular weight at 2,000,000 Da; purity ≈ 75%) was purchased from Jiahe Xuri Food Co., Ltd. (Henan Province, China). Glucose, fructose, xylose, and galactose were supplied by Shanghai Yuanye Bio-Technology Co., Ltd. (Shanghai, China). Tannic acid (TA) was provided by Aladdin Biochemical Technology Co., Ltd. (Shanghai, China). All other chemicals and reagents utilized were of chromatographic or analytical grade.

### 2.2 *In vitro* human fecal fermentation

Fresh fecal samples were donated by three healthy donors with a body mass index of 18.5–23.9, who had no known metabolic or gastrointestinal diseases and had not taken antibiotics and probiotic products for at least 3 months. Fresh feces were collected using the stool collectors (Sangon Biotech, Shanghai, China) and placed under anaerobic conditions (90% N₂, 5% CO₂, 5% H₂) within 15 min. Fresh feces samples from each donor were mixed and dissolved in sterile phosphate buffer (25%, w/w) by vortex and filtered through four-layer sterile medical gauze to obtain the fecal slurry. In vitro fermentation was conducted according to the protocol previously reported (Olano-Martin et al., 2000). According to our previous study (Liu et al., 2024b), 0.5 μmol/mL TA had no significant effect on the fermentation rate of resistant starch, and 1.5 μmol/mL TA had a stronger ability to decrease the fermentation rate of resistant starch than 1.0 μmol/mL TA. Moreover, 0.5–2.0 μmol/mL TA increased the total bacterial number. Therefore, the TA concentration was set as 1.5 μmol/mL. The concentration of dietary fibers was set up according to the previous report (He X. et al., 2023; He X. X. et al., 2023). The basic medium (1.0 L) was composed of peptone (2.0 g), yeast extract (2.0 g), NaHCO_3_ (2.0 g), L-cysteine hydrochloride (0.5 g), bile salts (0.5 g), NaCl (0.1 g), K_2_HPO_4_ (0.04 g), KH_2_PO_4_ (0.04 g), MgSO_4_·7H_2_O (0.01 g), CaCl_2_·H_2_O (0.01 g), haemin (5.0 mg), vitamin K_1_ (10.0 μL), Tween 80 (2.0 mL) and resazurin (1.0 mg). To initiate fermentation, 0.2 mL fecal slurry was added into 4.8 mL medium, which was the control group. The fermentation media with 1.5 μmol/mL TA or 10 mg/mL INU, FOS, XOS, GOS, IMO, and BG were used as positive control groups, which were named the TA group, the INU group, the FOS group, the XOS group, the GOS group, the IMO group, and the BG group, respectively. The fermentation media with mixtures of 1.5 μmol/mL TA and 10 mg/mL INU, FOS, XOS, GOS, IMO, or BG were named the TA_INU group, the TA_FOS group, the TA_XOS group, the TA_GOS group, the TA_IMO group, and the TA_BG group, respectively. Fermentation was carried out in a vibrating incubator (37 °C, 120 rpm). The fermentation samples were collected at 0, 3, 6, 12, 24, and 48 h and stored at −80 °C for further analysis.

On the other hand, glucose, fructose, xylose, and galactose were added into the basic media and used as positive control groups, which were named the Glu group, the Fru group, the Xyl group, and the Gal group, respectively. The fermentation media with mixtures of 1.5 μmol/mL TA and 10 mg/mL glucose, fructose, xylose, or galactose were named the TA_Glu group, the TA_Fru group, the TA_Xyl group, or the TA_Gal group, respectively.

### 2.3 Determination of gas production and pH

Gas was collected by syringes and its production was measured. Besides, the pH of the fermentation broth was measured using a standard pH meter (Mettler Toledo, China).

### 2.4 Determination of SCFAs

The concentration of acetate, propionate, and butyrate during fermentation was quantified by a gas chromatography system (7890B, Agilent Technologies, United States) equipped with an HP-FFAP capillary column (19091F-43, 30 m × 250 μm × 0.25 μm, Agilent Technologies, United States) and a flame ionization detector. The fermentation samples were centrifuged at 12,500 *g* for 5 min to obtain the supernatant. The supernatant was filtered using a 0.22-μm syringe filter, and 1 M HCl solution and 1 mM 2-ethylbutyric acid solution (the internal standard) were added. Subsequently, 1 μL treated sample was injected into the gas chromatography system. Helium was used as the carrier gas at a flow rate of 30 mL/min. The temperature was held at 60 °C for 5 min, then increased to 160 °C at a rate of 10 °C/min and held for 2 min, and increased to 220 °C at a rate of 20 °C/min. The injector and detector temperatures were maintained at 220 °C and 260 °C, respectively. Calibration curves were constructed using acetate, propionate, and butyrate standards, and concentrations were calculated based on response factors relative to 2-ethylbutyric acid.

### 2.5 Determination of the gallic acid concentration during fermentation

The fermentation broth supernatant was filtered using a 0.22-μm syringe filter, and the gallic acid concentration was detected using a liquid chromatography system with a UV detector (1,260, Agilent Technologies, United States). UV detection was performed at 280 nm. A Poroshell 120 SB-C18 column (4.6 × 150 mm, 4 μm; Agilent Technologies, United States) was used. The column temperature was maintained at 30 °C, and the injection volume was 5 μL. The mobile phase consisted of 0.1% phosphoric acid solution (Eluent A) and acetonitrile (Eluent B) at a flow rate of 0.8 mL/min. The gradient was established by mixing Eluent A (0–11 min, 80–50%; 11–17 min, 50–80%) and Eluent B (0–11 min, 20–50%; 11–17 min, 50–20%).

### 2.6 Analysis of the activity of glycoside hydrolases

Tannic acid (1.5 μmol/mL) was added into the control fermentation broth, and the activity of glycoside hydrolases before and after the addition of tannic acid was determined. The *β*-glucanase, xylanase, *α*-glucosidase, and *β*-galactosidase activity of the control group was determined using the *β*-glucanase activity assay kit (K-MBGL; Megazyme International Ireland, Bray, Ireland), xylanase activity assay kit (T-XAX; Megazyme International Ireland, Bray, Ireland), *α*-gucosidase activity assay kit (Beijing Solarbio Science & Technology Co., Ltd.) and *β*-galactosidase activity assay kit (Beijing Solarbio Science & Technology Co., Ltd.) according to the manufacturers’ protocols.

Due to lack of commercially available assay kits, the *β*-fructosidase activity was assessed by monitoring the fructose concentration during fermentation (Paludan-Müller et al., 2002). The fructose concentration was determined using a Dionex ICS-6000 ion chromatography system (Thermo Fisher Scientific, Waltham, MA, United States). The system consisted of a dual-piston pump, an AS-SP autosampler, and a DC electrochemical detector. The electrochemical detector employed an Ag/AgCl reference electrode and a gold working electrode, applying the standard carbohydrate quadrupole waveform. A CarboPac™ PA20 guard column (3.0 mm × 30 mm, Thermo Fischer Scientific, Waltham, MA, United States) and a CarboPac™ PA20 analytical column (3.0 × 150 mm, Thermo Fischer Scientific, Waltham, MA, United States) were used. The supernatant was filtered through a 0.22-μm syringe filter, and 25 μL sample was injected. The mobile phase was composed of ultrapure water (Eluent A), 10 mmol/L NaOH solution (Eluent B), 250 mmol/L NaOH solution (Eluent C), and 500 mmol/L CH_3_COONa solution (Eluent D). The gradient was established by mixing Eluent B (0–20 min, 10%; 20–30 min, 20%; 48–60 min, 10%), Eluent C (30–48 min, 80%) and Eluent D (20–30 min, 10–40%), supplemented by Eluent A to 100%. The temperature was set as 30 °C, and the flow rate was 0.5 mL/min. Chromeleon 7 software was utilized for data processing. The fructose concentration in fermentation broth was quantified using a calibration curve by comparing the peak area to that of standard solutions.

### 2.7 Analysis of 16S rRNA gene sequences

DNA was extracted from the fecal fermentation slurry, and its quantity and quality were evaluated using UV spectrophotometry and agarose gel electrophoresis. Absorbance at 260 nm and 280 nm was measured to calculate the DNA concentration. Generally, an A260/A280 ratio of 1.8–2.0 indicates high purity. Additionally, DNA was separated via agarose gel electrophoresis, and bands were visualized using a gel imaging system. Clear and intact bands confirmed DNA suitability for subsequent experimental applications. The V3-V4 region of the 16S rRNA gene was amplified from each sample using polymerase chain reaction (PCR). The amplification was performed with the forward primer 338F (5’-ACTCCTACGGGAGGCAGCA-3′) and the reverse primer 806R (5’-GGACTACCAGGGTATCTAAT-3′). The amplicons were quantified using the PicoGreen dsDNA Assay Kit to ensure accurate measurement of the DNA concentration. Following individual quantification, equimolar amounts of amplicons were pooled to create a normalized library for sequencing. After the individual quantification step, amplicons were pooled in equal amounts, and pair-end 2,250 bp sequencing was performed using the Illlumina NovaSeq platform with NovaSeq 6,000 SP Reagent Kit (500 cycles) at Shanghai Personal Biotechnology Co., Ltd. (Shanghai, China). Microbiome bioinformatics were performed with QIIME2 2022.11. Briefly, raw sequence data were demultiplexed using the demux plugin following by primers cutting with cutadapt plugin. Sequences were then quality filtered, denoised, merged and chimera removed using the DADA2 plugin. Non-singleton amplicon sequence variants (ASVs) were aligned with mafft and used to construct a phylogeny with fasttree2. Alpha-diversity metrics, including Chao1, observed species, Shannon, Simpson, Faith’s PD, Pielou’s evenness and Good’s coverage, and beta diversity metrics, including weighted UniFrac, unweighted UniFrac, Jaccard distance, and Bray–Curtis dissimilarity, were estimated using the diversity plugin with samples were rarefied to 35,891 sequences per sample. Taxonomy was assigned to ASVs using the classify-sklearn naïve Bayes taxonomy classifier in feature-classifier plugin against the Greengenes 13.8 Database. The resulting sequencing data were analyzed to characterize the microbial composition and to assess both *α*-diversity (within-sample diversity) and *β*-diversity (between-sample diversity) based on operational taxonomic units (OTUs).

### 2.8 Statistical analysis

**Figure 1:**
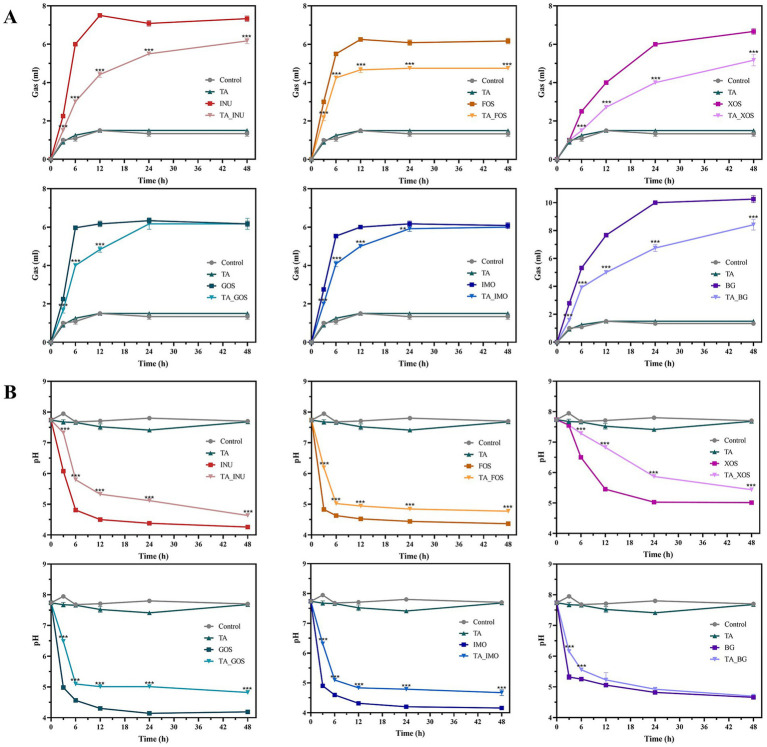
Gas production **(A)** and pH **(B)** from fermentation of dietary fibers. **p < 0.01, ***p < 0.001 for TA_ dietary fiber groups vs. dietary fiber groups at each time point. The interaction effect was significant (*p* < 0.001).

**Figure 2:**
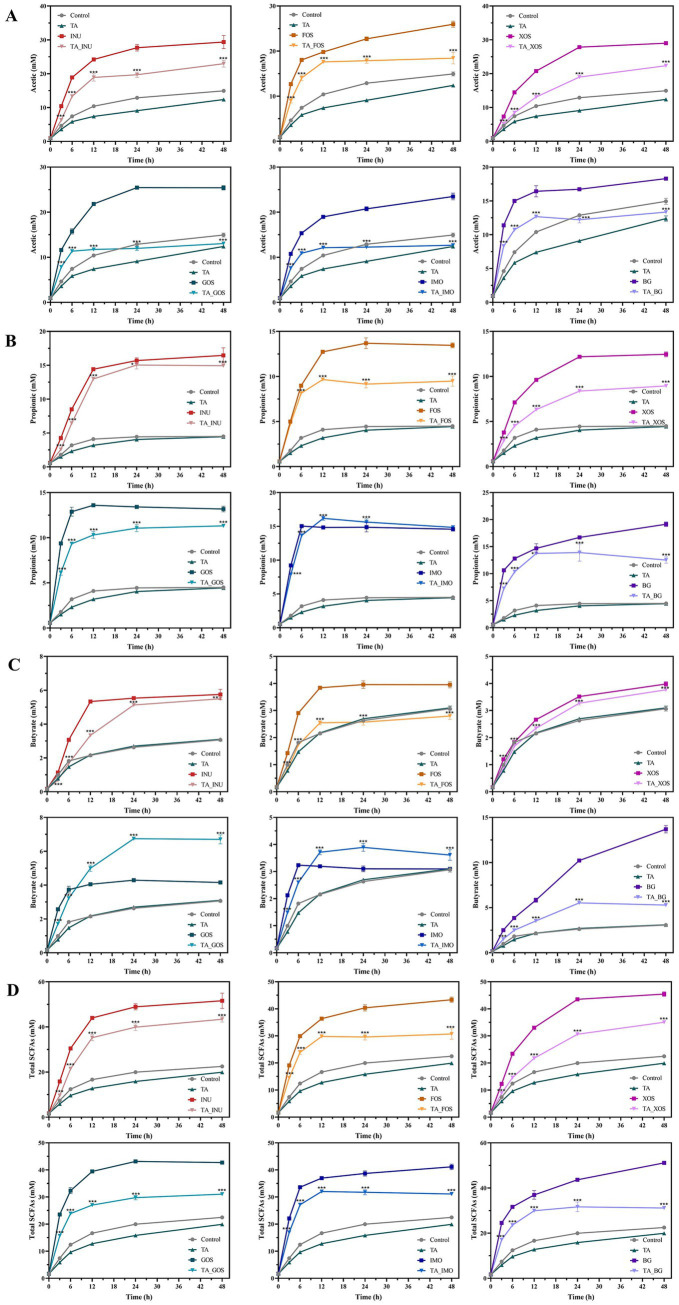
Production of acetate **(A)**, propionate **(B)**, butyrate **(C)**, and total SCFAs **(D)** from fermentation of dietary fibers. ^*^p < 0.05, **p < 0.01, ***p < 0.001 for TA dietary fiber groups vs. dietary fiber groups at each time point. The interaction effect was significant (*p* < 0.001).

**Figure 3:**
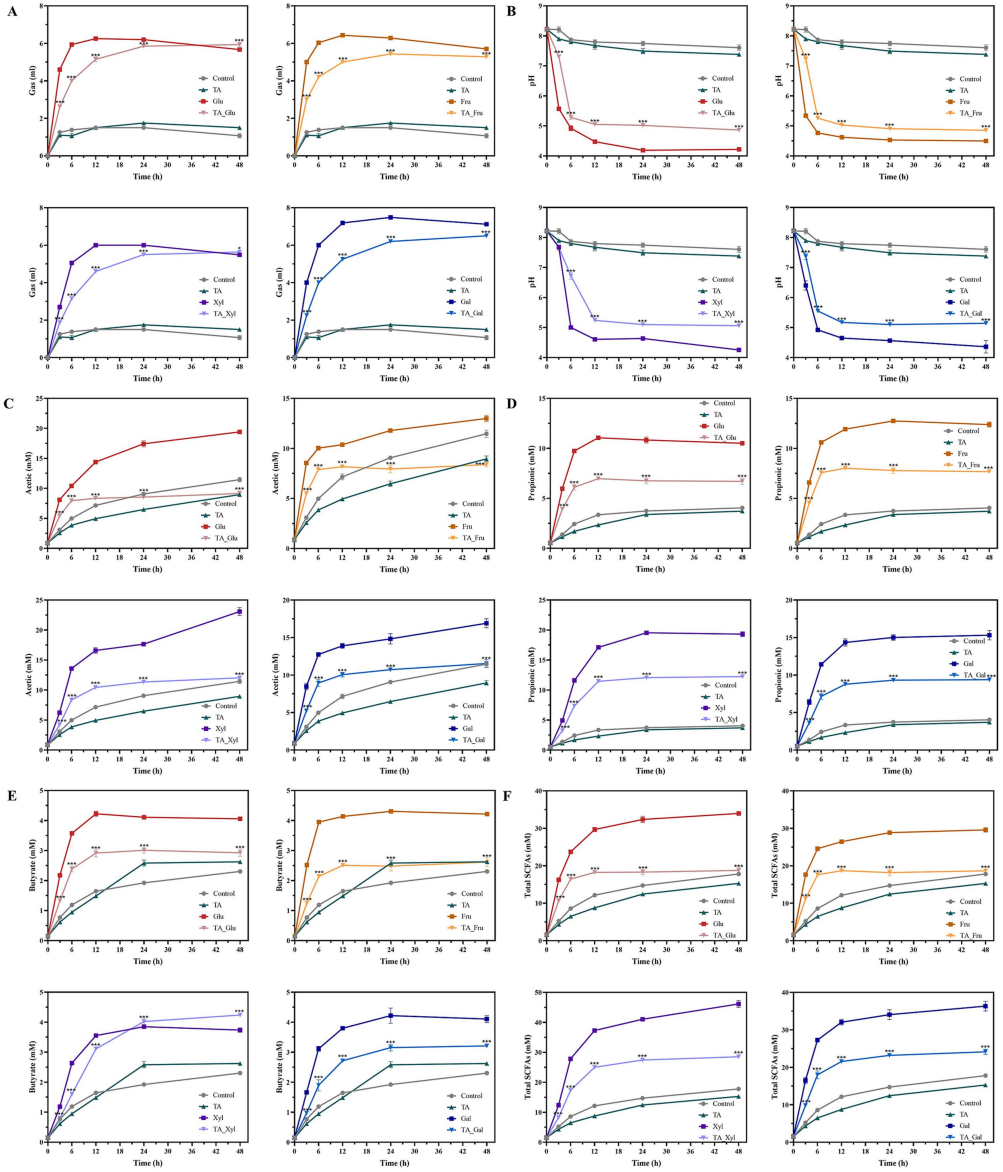
The fructose concentration in groups containing INU **(A)** and FOS **(B)** during fermentation and the xylanase **(C)**, *β*-galactosidase **(D)**, *α*-glucosidase **(E)**, and *β*-glucanase **(F)** activity before and after addition of TA. Values with different letters are statistically different from each other (*p* < 0.05).

**Figure 4:**
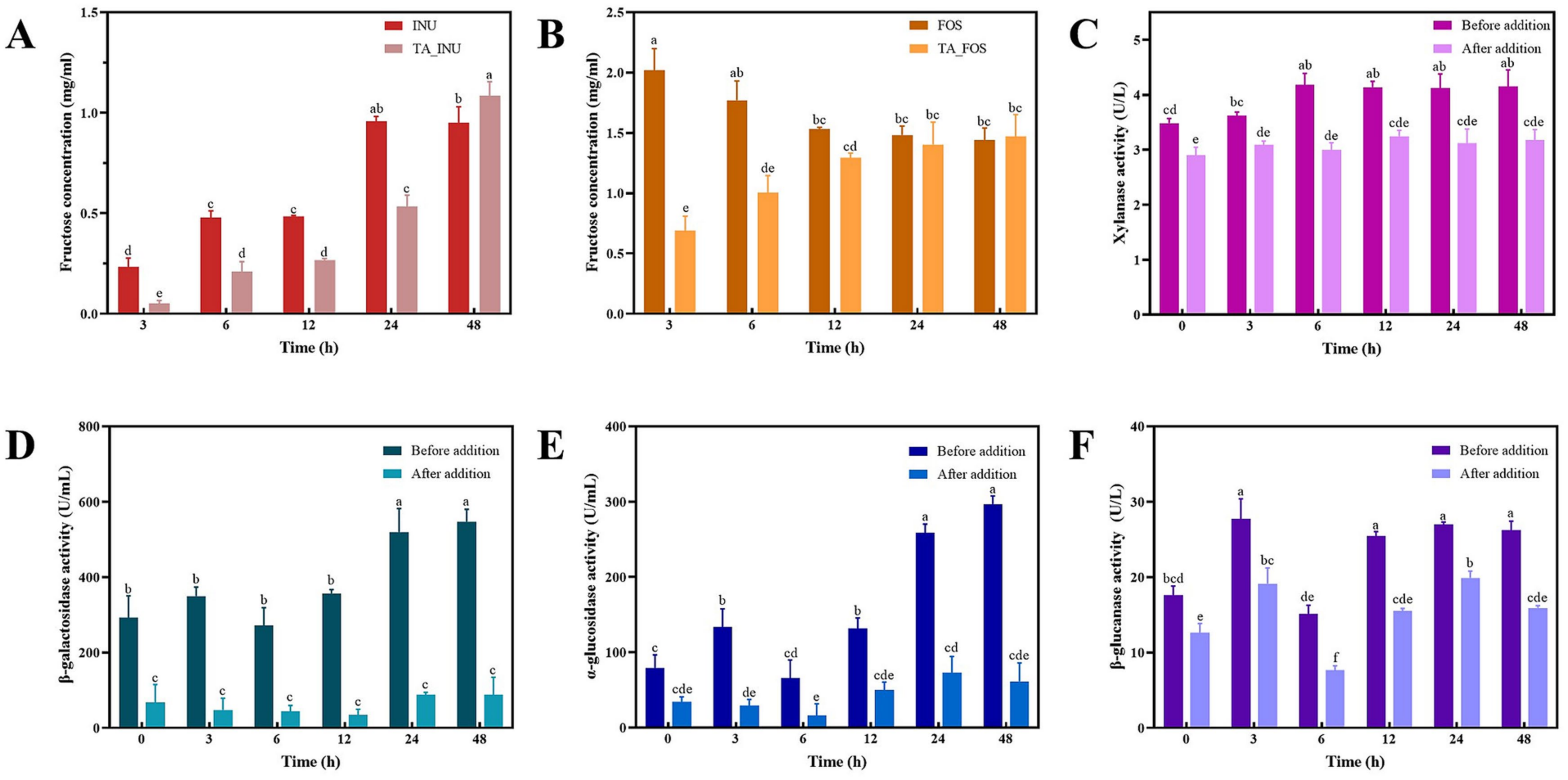
Gas production **(A)**, pH **(B)**, production of acetate **(C)**, propionate **(D)**, butyrate **(E)**, and total SCFAs **(F)** from fermentation of monosaccharides. *p < 0.05, ***p < 0.001 for TA_monosaccharide groups vs. monosaccharide groups at each time point. The interaction effect was significant (*p* < 0.001).

**Figure 5:**
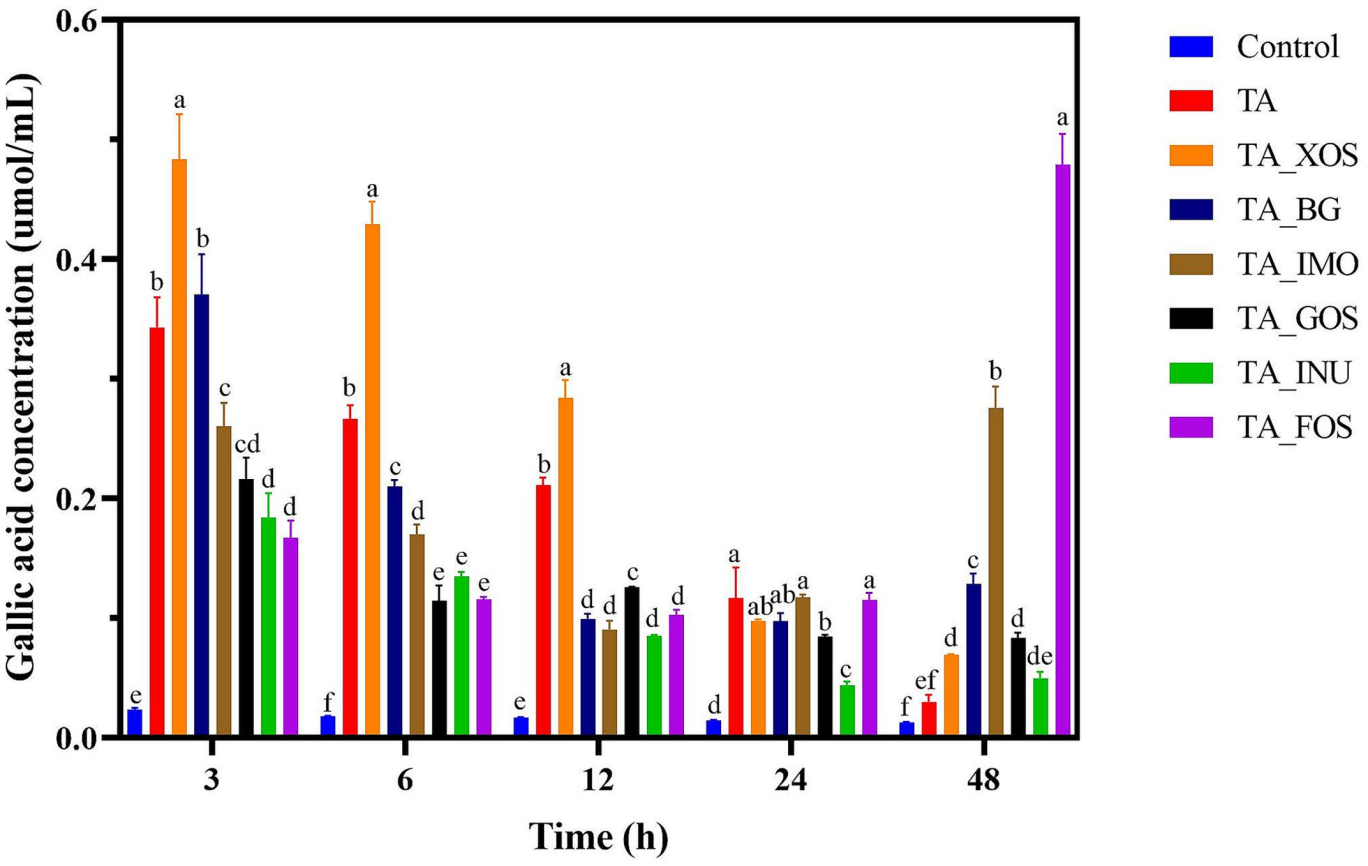
The gallic acid concentration during fermentation. Different letters were significantly different (*P* < 0.05).

All tests in this study were performed at least in triplicate, and data are expressed as the mean ± standard deviation. Statistical analyses and graphical displays were performed using SPSS Statistics 26.0 (SPSS, Inc., Chicago, Illinois) and GraphPad Prism 9.0 (GraphPad Software, Inc., La Jolla, California). Data in Figures 1–3 were analyzed by two-way analysis of variance (ANOVA), and data in Figures 4, 5 were analyzed by one-way ANOVA. Statistical significance was established at *p* < 0.05. Statistical analysis of microbiota was performed using R^1^.

## 3 Results

### 3.1 Effect of TA on gas production, pH, and production of SCFAs during fermentation of dietary fibers

There was no significant difference in gas production, pH, and production of SCFAs between the TA group and the control group (Figures 1, 2). Groups containing dietary fibers exhibited significantly higher gas and SCFA production and lower pH values than the control group. Moreover, the gas and SCFA production of groups containing INU, FOS, XOS, GOS, IMO, or BG increased rapidly from 0 to 6 h and the pH decreased rapidly, suggesting that these dietary fibers were metabolized rapidly. Notably, the TA/dietary fiber mixture groups had lower gas and SCFA production and higher pH than groups supplemented with only FOS, INU, XOS, GOS, IMO, or BG, which demonstrate that the addition of 1.5 μmol/mL TA effectively reduced the fermentation rate of these dietary fibers.

Moreover, the supplementation of TA exerted differential effects on the production of individual acetate, propionate, and butyrate from fermentation of different dietary fibers. Specifically, the addition of TA decreased the acetate, propionate and butyrate production from fermentation of INU, FOS, XOS and BG throughout the entire fermentation. TA led to a decrease in the production of acetate and propionate from fermentation of GOS. However, the butyrate production showed a significant increase at the late stage of fermentation (24–48 h). Accordingly, the TA_GOS group had more butyrate than the GOS group. This result might be due to that TA promoted the growth of butyrate-producing bacteria during fermentation of GOS. Analogously, the TA_IMO group had less acetate and more butyrate and propionate than the IMO group at the late stage of fermentation. The effect of TA on fermentation of resistant starch was different from those described above (Liu et al., 2024b). Similarly, rutin altered patterns of SCFA production from fermentation of pectin, psyllium, and resistant maltodextrin (Havlik et al., 2020). However, purple sweet potato polyphenols had no impact on SCFA production from fermentation of INU (Kilua et al., 2019). These differences might be associated with the different effects of polyphenol/dietary fiber on the composition of gut microbiota and the different polyphenol concentrations.

### 3.2 Impact of TA on the glycoside hydrolase activity of gut microbiota and fermentation of monosaccharides

As stated above, we previously confirmed that TA reduced the fermentation rate of resistant starch, because TA inhibited the amylase activity of gut bacteria. It has been reported that polyphenols can also inhibit the activity of various enzymes except amylases, such as protease, glucosidase, phospholipases A2, and lipase (El-Saadony et al., 2024; He X. et al., 2023; Kan et al., 2022). The inhibitory effect of polyphenols on the activity of enzymes mainly results from the binding interaction between polyphenol molecules and enzyme molecules (Miao, et al., 2021; Yu et al., 2024). It is possible that this interaction widely exists between polyphenol molecules and protein molecules. Thus, it is inferred that TA was able to inhibit glycoside hydrolases that hydrolyzed fibers used in this work and thus decrease the fermentation rate of these fibers. On the other hand, monosaccharides resulting from hydrolysis of dietary fibers are further metabolized to produce SCFAs, which also involves enzymes, such as hexokinase, phosphofructokinase and pyruvate kinase. In other words, TA might also inhibit the activity of these enzymes and decrease the fermentation rate of dietary fibers. Accordingly, the effect of TA on the activity of *β*-fructosidase, xylanase, *β*-galactosidase, *α*-glucosidase, and *β*-glucanase was determined, and the effect of TA on fermentation of monosaccharides, including glucose, fructose, xylose, and galactose, was studied.

*β*-fructosidase hydrolyzes INU or FOS to release fructose. Thus, the *β*-fructosidase activity was assessed by the fructose concentration. During fermentation, the fructose concentration in the INU group gradually increased (Figure 3A), indicating that INU was gradually hydrolyzed by *β*-fructosidase secreted by gut microbiota to release free fructose. The fructose concentration in the FOS group reached the peak value after fermentation for 3 h and then slightly decreased (Figure 3B), which suggests that FOS was rapidly hydrolyzed into fructose and metabolized to SCFAs by gut bacteria. This metabolic difference of FOS and INU might be due to lower degree of polymerization of FOS than INU. It is noteworthy that at the early stage of fermentation (0–6 h), the TA_INU group or the TA_FOS group had lower fructose concentration than the group containing INU or FOS alone. The fructose concentration in the TA_INU group or the TA_FOS group increased progressively during fermentation and was similar to that of the INU group or the FOS group after fermentation for 48 h. These results demonstrate that TA effectively inhibited the *β*-fructosidase activity. Similarly, the addition of TA significantly decreased the activity of xylanase, *β*-galactosidase, *α*-glucosidase, and *β*-glucanase (Figures 3C–F). These results confirm that TA inhibited the activity of enzymes that participated in the first fermentation stage of dietary fibers.

The gas production in monosaccharide groups increased rapidly during the first 6 h (Figure 4A), indicating that these monosaccharides were rapidly metabolized. In the TA/monosaccharide mixture groups, the gas production was significantly reduced. Concurrently, the addition of TA decreased acid production from fermentation of monosaccharides. During fermentation, the pH of the TA/monosaccharide mixture groups was higher than that of groups with only monosaccharides (Figure 4B). Regarding SCFA generation, the groups containing monosaccharides demonstrated rapid accumulation of total SCFAs during the first 6 h, and the addition of TA substantially inhibited production of total SCFAs. Specifically, production of acetate, propionate, butyrate, and total SCFAs in TA/monosaccharide mixture groups was lower than that of groups with only monosaccharides (Figures 3C–F). These results indicate that TA slowed down fermentation of monosaccharides. Thus, it is inferred that TA also inhibited the activity of enzymes that participated in the second fermentation stage of dietary fibers. As a result, TA slowed down fermentation of dietary fibers. In summary, TA slowed down the whole metabolic process of dietary fibers due to its inhibitory effect on universal enzymes.

### 3.3 The metabolism of TA

The TA is hydrolyzed into gallic acid, and gallic acid is further metabolized into urolithin by gut microbiota (Loo et al., 2020; Nakamura et al., 2003). Consequently, the gallic acid concentration was examined to assess effects of dietary fibers on the metabolic pattern of TA. The gallic acid concentration in the TA group reached the peak value at 3 h and then decreased (Figure 5), indicating that TA was rapidly hydrolyzed by gut microbiota. The gallic acid concentration in TA_INU, TA_XOS, TA_GOS, and TA_BG groups also reached the peak value at 3 h. Besides, the gallic acid concentration at 3 h was higher in the TA_XOS group and the TA_BG group than in the TA group, indicating that XOS and BG promoted the hydrolysis degree of TA. In contrast, the TA_INU group and the TA_GOS group exhibited lower gallic acid concentration than the TA group, suggesting that INU and GOS reduced the hydrolysis degree of TA. Particularly, the gallic acid concentration reached the peak value at 48 h in the TA_FOS group and the TA_IMO group, which suggests that FOS and IMO significantly slowed down the metabolism of TA. Thus, it is suggested that dietary fibers affected the metabolism of TA, depending on the dietary fiber type. Yang et al. (2021) found that the addition of FOS slowed down the degradation of cyanidin-3-*O*-glucoside, and the mixture group had lower abundance of *Fusobacterium* than the cyanidin-3-*O*-glucoside group. Moreover, it was reported that RG-I pectic polysaccharides modulated the metabolic of hesperidin by promoting the growth of *Blautia*, *Faecalibacterium*, and *Prevotella* (Wu et al., 2025). Therefore, it was inferred that XOS and BG promoted growth of TA-metabolizing bacteria and the degradation of TA. Moreover, other four dietary fibers inhibited the growth of TA-metabolizing bacteria, thus inhibiting or slowing down the degradation of TA.

### 3.4 Analysis of microbial composition

#### 3.4.1 Diversity of gut microbiota

The INU and FOS were selected as representatives of dietary fibers, and the effect of interplay between TA and INU or FOS on the composition of gut microbiota was analyzed. The *α*-diversity and *β*-diversity of the gut microbial community were calculated based on 97% similarity threshold of OTUs. To comprehensively evaluate the composition of the microbial community, the Shannon index and the Chao1 index were employed to reflect the diversity and richness. In this study, fermentation for 3 h was used to represent the early fermentation period, and fermentation for 24 h was used to represent the late fermentation period. After fermentation for 3 h, the Shannon index was significantly lower in all experimental groups than the control group (*p* < 0.05) (Table 1), which suggests that the addition of TA, INU and FOS reduced the diversity of the bacterial flora. Although no significant differences were observed (*p* > 0.05), the Chao1 index of the TA_FOS group or the TA_INU group were lower than the FOS group or the INU group. These findings imply that the addition of TA reduced the abundance and diversity of gut microbiota at the early fermentation stage, probably due to slowing down the utilization of FOS and INU. The Shannon index and the Chao 1 index of each group after fermentation for 24 h were lower than those after fermentation for 3 h (Table 2), which might be due to the substrate consumption and metabolite accumulation. The Shannon index of the TA group was significantly higher than that of the control group (*p* < 0.05). The group containing TA/FOS or TA/INU had higher Shannon index than the group containing FOS or INU alone (*p* < 0.05). Furthermore, although no significant differences were observed (*p* > 0.05), the Chao1 index of the TA_FOS group or the TA_INU group was higher than the FOS group or the INU group. These results indicate that the mixture of TA and FOS or INU enhanced microbial diversity and richness at the late fermentation stage. Thus, it is inferred that the addition of TA prolonged fermentation of FOS and INU. As a result, more fibers were available at the late fermentation stage, and the mixture groups exhibited higher Chao1 index and Shannon index than groups containing INU or FOS alone after fermentation for 24 h.

**Table 1:** The *α*-diversity after fermentation for 3 h.

Group	*α*-diversity	
	Shannon	Chao 1
Control	6.77 ± 0.12^a^	666.05 ± 95.40^a^
TA	6.45 ± 0.05^b^	567.97 ± 94.34^a^
FOS	6.37 ± 0.04^bc^	730.81 ± 59.77^a^
TA_FOS	6.33 ± 0.04^bc^	650.39 ± 66.91^a^
INU	6.20 ± 0.09^c^	664.77 ± 92.61^a^
TA_INU	6.26 ± 0.08^bc^	632.27 ± 95.69^a^

Mean values with different letters in the same column are significantly different at p < 0.05.

**Table 2:** The *α*-diversity after fermentation for 24 h.

Group	*α*-diversity	
	Shannon	Chao 1
Control	6.15 ± 0.03^a^	592.84 ± 42.01^a^
TA	6.30 ± 0.11^a^	380.64 ± 75.38^b^
FOS	5.35 ± 0.06^d^	438.94 ± 16.79^b^
TA_FOS	6.24 ± 0.04^a^	518.65 ± 53.94^ab^
INU	5.72 ± 0.06^c^	442.68 ± 29.67^b^
TA_INU	5.89 ± 0.05^b^	472.49 ± 64.35^ab^

Mean values with different letters in the same column are significantly different at *p* < 0.05.

**Figure 6:**
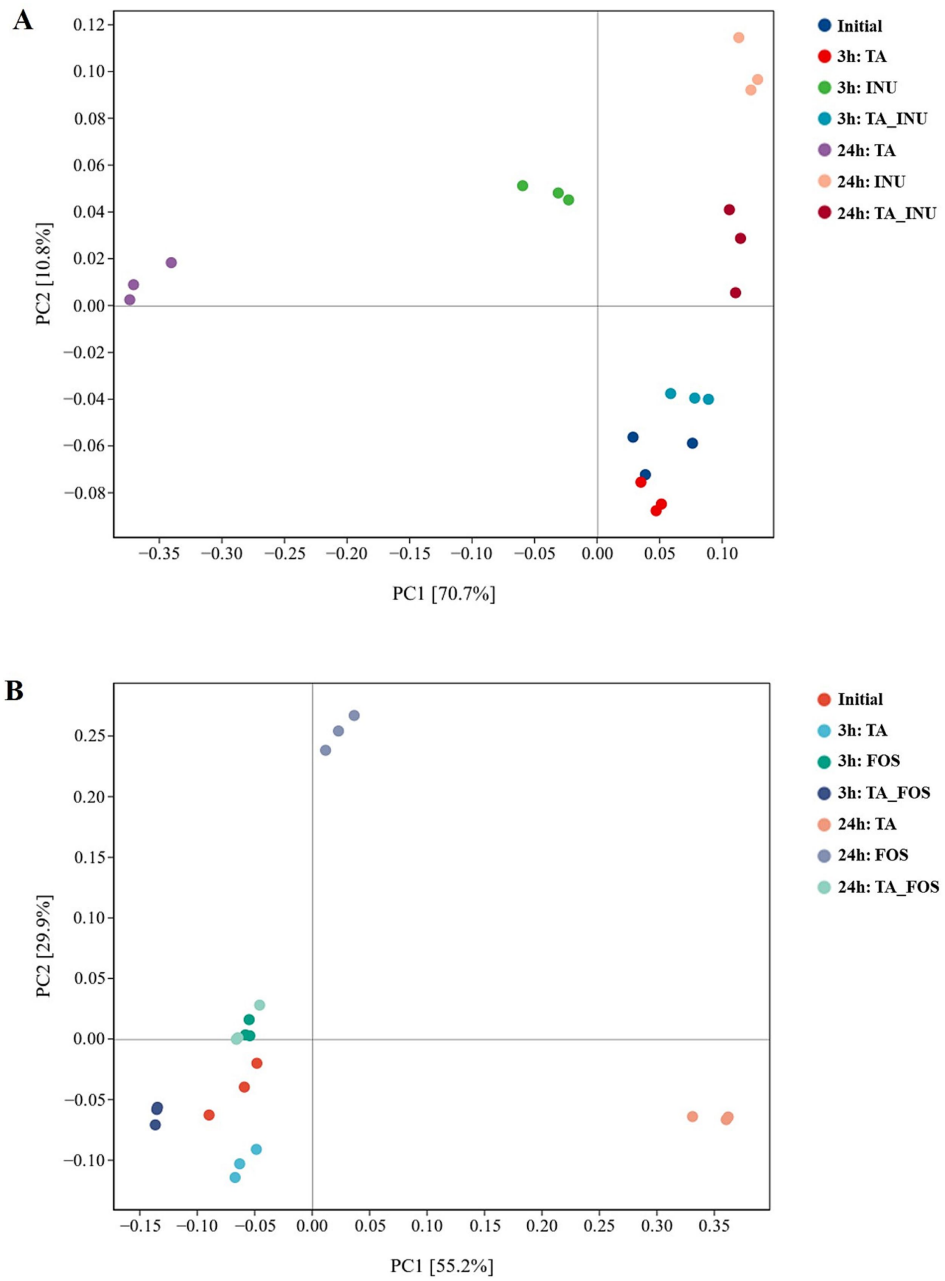
Principal component analysis (PCA) of *β*-diversity in groups containing INU **(A)** and containing FOS **(B)** after fermentation for 3 h and 24 h.

The *β*-diversity was analyzed according to the relative abundance of OTUs at 3 h and 24 h based on Weighted Unifrac distance (Figures 6A,B). After fermentation for 3 h, the microbial composition of the TA group, the FOS group, and the INU group gradually separated with the control group. As fermentation progressed for 24 h, a significant separation was observed between the control group and the TA group, the FOS group or the INU group, indicating that TA, FOS and INU significantly changed the microbial composition. The TA_INU group showed significant separation with the TA group or the INU group, indicating that the effect of the TA and INU mixture on the microbial composition was different from that of TA or INU alone (Figure 6A). Moreover, the microbial composition of the TA_FOS group at 24 h was similar to that of the FOS group at 3 h (Figure 6B). These observations suggest that the addition of TA delayed the impact of INU and FOS on the microbial composition, and the effect of TA on fermentation of FOS was stronger than that on fermentation of INU, which might be due to that FOS had lower degree of polymerization than INU.

#### 3.4.2 Microbial composition at the genus level

**Figure 7:**
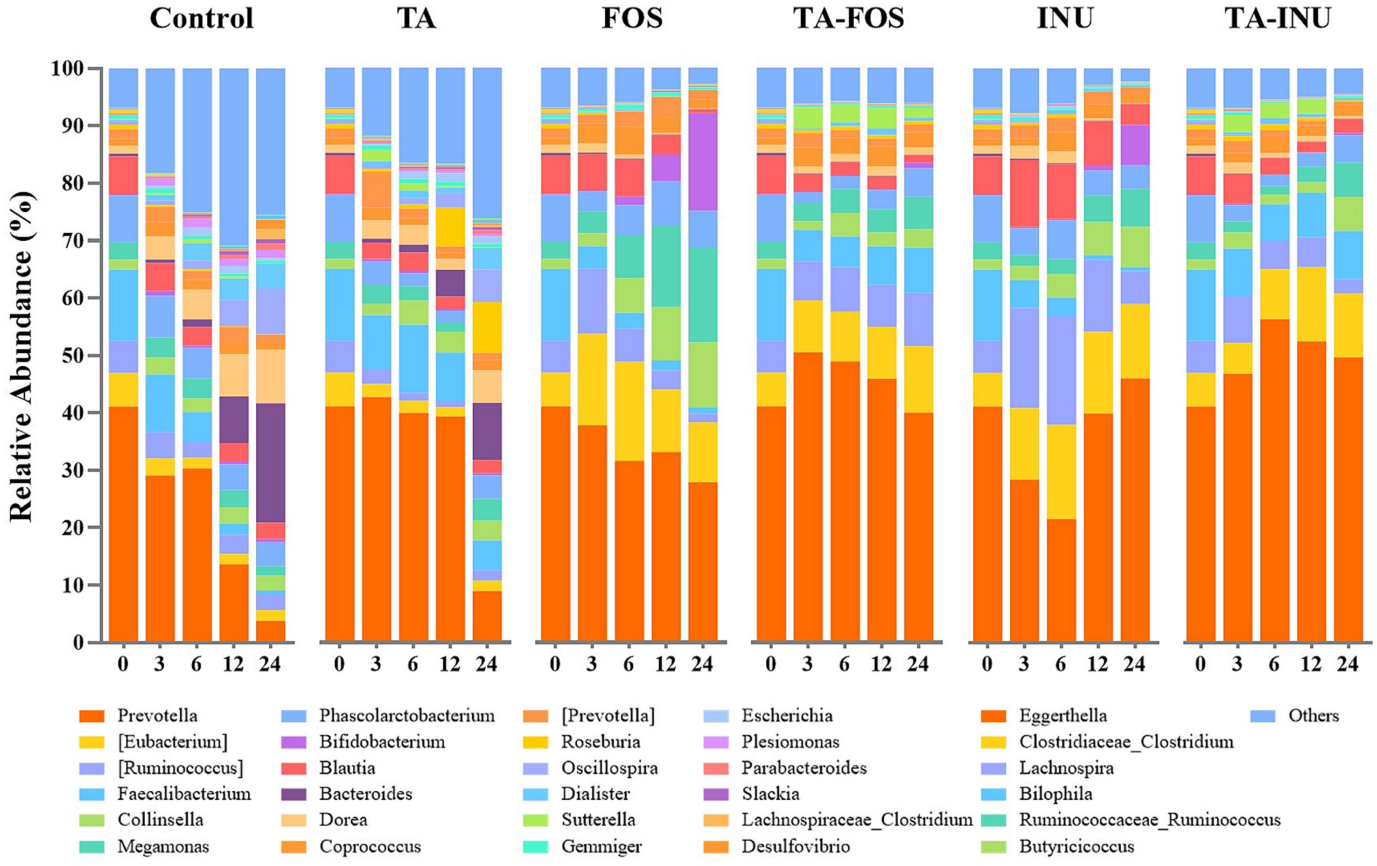
Relative abundance of the bacterial genera in INU-containing and FOS-containing groups.

The relative abundance of *Eubacterium* and *Ruminococcus* in the FOS group and the INU group increased significantly during the first 3 h and decreased from 6 to 24 h (Figure 7). It has been documented that FOS and INU promote the growth of *Eubacterium* and *Ruminococcus* (Hindle et al., 2025; Mao et al., 2015). Thus, it is inferred that the rapid fermentation of FOS and INU stimulated the proliferation of these bacteria. As FOS and INU were rapidly consumed, the abundance of these bacteria gradually declined. Interestingly, the relative abundance of *Eubacterium* and *Ruminococcus* in the TA_FOS group gradually increased during fermentation. In the TA_INU group, the relative abundance of these bacteria increased gradually during the first 12 h and then decreased. These results aligned with effects of INU and FOS on the metabolic pattern of TA. Thus, it is inferred that TA only inhibited fermentation of INU at the early stage, because the gallic acid concentration reached the peak value at 3 h in the TA_INU group. After TA was metabolized, the *β*-fructosidase activity was recovered, and INU was metabolized at the late stage. In the TA_FOS group, the gallic acid concentration reached the peak value at 48 h. As a result, TA was continuously at high concentration and inhibited fermentation of FOS. Accordingly, the relative abundance of these bacteria gradually increased. In summary, the above results also indicate that the addition of TA prolonged fermentation of FOS and INU, and TA exerted a more profound effect on FOS.

In the FOS group and the INU group, the relative abundance of *Prevotella* decreased from 0 to 6 h. This reduction might be due to that the rapid fermentation of FOS and INU lowered the pH value and inhibited the growth of *Prevotella* (Tanner et al., 2011; Walker et al., 2005). The abundance of *Prevotella* was higher in the mixture groups than in groups containing FOS or INU alone during fermentation. This might be due to that addition of TA slowed down fermentation of FOS and INU and the mixture groups had higher pH. FOS and INU are known to selectively stimulate the growth of *Bifidobacterium* (Nagy et al., 2023). In the FOS group, the relative abundance of *Bifidobacterium* gradually increased and reached 16.9% at 24 h. Particularly, *Bifidobacterium* were enriched at 12 h. However, *Bifidobacterium* were enriched at 24 h in the INU group, and its relative abundance reached 7.02% at 24 h. The different effects of INU and FOS on the relative abundance of *Bifidobacterium* might be due to their different chain length. Although *Bifidobacterium* are generally considered to be beneficial, their excessive proliferation may break intestinal microbial balance (Dey, 2024). For instance, supplementation of FOS and GOS inhibited the growth of butyrate-producing microbes and deteriorated glucose metabolism, because they dramatically increased the abundance of *Bifidobacterium* (Liu et al., 2017). The relative abundance of *Bifidobacterium* in the TA_FOS group and the TA_INU group reached 1.05 and 0.39% at 24 h, respectively. These results also indicate that TA slowed down the metabolism of FOS and INU. Moreover, mixing with TA inhibited excessive proliferation of *Bifidobacterium* induced by fermentation of FOS and INU, probably exhibiting more benefits. Furthermore, fermentation of TA increased the relative abundance of *Faecalibacterium*, a pivotal probiotic in the gut that enhances intestinal barrier function, modulates the immune response, and reduces inflammation (Machiels et al., 2014; Martín et al., 2023; Miquel et al., 2013; Mohebali et al., 2023). In comparison, fermentation of FOS and INU resulted in a continuous decrease in the relative abundance of *Faecalibacterium*. The relative abundance of *Faecalibacterium* in the mixture groups remained higher than in groups containing FOS or INU and gradually increased. Thus, it is inferred that the regulation of TA on the microbial composition also contributed to that TA slowed down fermentation of FOS and INU. Similarly, it was reported that mixing different dietary fibers decreased the fermentation rate, because different dietary fibers selectively promoted the growth of different specific genera and these genera competed to proliferate (Cantu-Jungles et al., 2025; Yoshida et al., 2024; Zhang Y. W. et al., 2024). Additionally, the relative abundance of *Megamonas* and *Collinsella* increased significantly in FOS and INU groups at the late stage of fermentation. Specifically, supplement of FOS increased the relative abundance of *Megamonas and Collinsella by* 445 and 535%, respectively, and supplement of INU increased the relative abundance of *Megamonas and Collinsella by* 117 and 296%, respectively. *Megamonas* may induce the colon cancer, and the high abundance of *Collinsella* leads to dysbiosis of gut microbiota (Gou et al., 2023; Yachida et al., 2019; Zhang L. Y. et al., 2024). Co-fermentation of TA and FOS or INU suppressed proliferation of these genera. Specifically, mixing TA and FOS increased the relative abundance of *Megamonas* and *Collinsella* by 87 and 81%, respectively, and mixing TA and INU increased the relative abundance of *Megamonas* and *Collinsella* by 97 and 235%, respectively. These results indicated that the mixtures had more healthy benefits than FOS or INU alone. In summary, these results confirm that TA modified the relative abundance of specific genera and regulated the microbial community dynamics, thus slowing down fermentation of FOS and INU and improving their prebiotic activity.

## 4 Discussion

**Figure 8:**
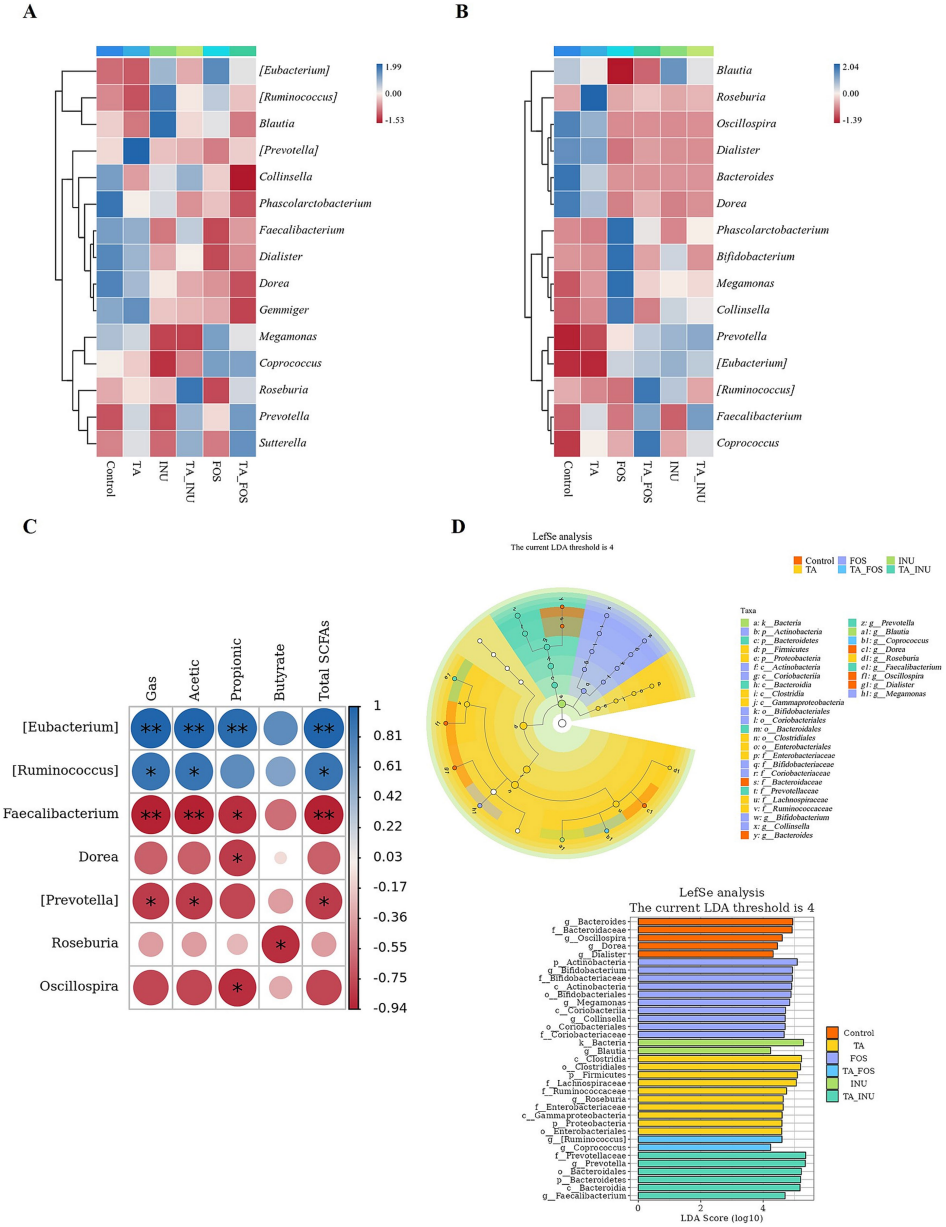
Heatmap of the composition of gut microbiota at 3 h **(A)** and 24 h **(B)**, Spearman correlation heatmap between the composition of gut microbiota and production of metabolites at 3 h **(C)**, and linear discriminant analysis effect size (LEfSe) and linear discriminant analysis (LDA) of microbiota at 24 h **(D)**. Blue and red indicated the positive correlation and negative correlation, respectively. *p < 0.05, **p < 0.01.

It was confirmed that TA decreased the fermentation rate of these dietary fibers due to the inhibitory effect on microbial enzymes. To confirm the mechanism of microbial community dynamics, correlation between the composition of gut microbiota and production of metabolites was established. At the initial stage of fermentation, the amount of metabolites could be used to indicate the fermentation rate. However, as fermentation progressed over a longer period, due to the accumulation of metabolites during *in vitro* fermentation, the amount of metabolites was not suitable for representing the fermentation rate. Thus, the spearman correlation between the microbial composition and the fermentation rate, as indicated by production of gas and SCFAs at 3 h, was established. After fermentation for 3 h, the fermentation rate was positively correlated with the abundance of *Eubacterium* and *Ruminococcus* that was lower in the mixture groups than in groups containing FOS or INU alone (Figures 8A,C). Meanwhile, the fermentation rate was negatively correlated with the relative abundance of *Faecalibacterium*, *Roseburia*, and *Prevotella* that was higher in groups containing TA_FOS or TA_INU than in groups containing FOS or INU. These results further indicate that the addition of TA suppressed the growth of genera that degraded and utilized FOS and INU, which also contributed to decreasing the fermentation rate. After fermentation for 24 h, the relative abundance of *Roseburia*, *Faecalibacterium*, and *Coprococcus* was higher in the TA group than in the control group (Figure 8B). These bacteria have been reported to inhibit the growth of pro-inflammatory bacteria and produce SCFAs (Kang et al., 2023; Machiels et al., 2014; Yang et al., 2023). The relative abundance of *Phascolarctobacterium*, *Bifidobacterium*, *Megamonas*, and *Collinsella* was higher in the FOS group than in the control group. The relative abundance of *Blautia*, *Bifidobacterium*, *Megamonas, Collinsella*, *Prevotella*, *Eubacterium* and *Ruminococcus* was higher in the INU group than in the control group. Notably, the co-fermentation of TA and FOS or INU significantly promoted the growth of *Faecalibacterium*, and *Coprococcus*. *Faecalibacterium* and *Coprococcus* are highly associated with the production of propionate and butyrate (Dicks, 2024; Huang et al., 2022; Zhou et al., 2018). These results also showed that mixing TA and FOS or INU selectively promoted the growth of beneficial bacteria and inhibited the growth of potentially harmful bacteria, exhibiting stronger prebiotic activity than TA or dietary fibers alone.

LEfSe analysis (LDA > 4, *p* < 0.05) showed that the addition of TA significantly changed the microflora characteristics during fermentation of INU or FOS and formed unique biomarkers (Figure 8D). At the genus level, the TA group exhibited higher abundance of *Roseburia,* which belong to butyrate-producing bacteria. The FOS group specifically enriched *Megamonas*, *Collinsella,* and *Bifidobacterium*. In the INU group, *Blautia* were dominant. In the TA_FOS group, *Ruminococcus* and *Coprococcus* became the signature bacteria, both of which are involved in complex polysaccharide metabolism. In the TA_INU group, *Prevotella* and *Faecalibacterium* were enriched. These results also indicate that the addition of TA significantly changed the microbial composition induced by FOS or INU. In summary, tannic acid altered the microbial composition, which also contributed to the decrease in the fermentation rate of FOS and INU.

## 5 Conclusion

Tannic acid reduced the fermentation rate of inulin, fructooligosaccharides, xylo-oligosaccharides, galacto-oligosaccharides, isomalto-oligosaccharides, and *β*-glucan, partially because tannic acid inhibited the activity of participated in the second fermentation stage of dietary fibers. Thus, it is inferred that polyphenols may be able to slow down fermentation of all types of dietary fibers. On the other hand, tannic acid slowed down the growth of dietary fiber-metabolizing bacteria, which also contributed to the decrease in the fermentation rate. In summary, co-supplementation of polyphenols and dietary fibers is an effective approach to decrease the fermentation rate of dietary fibers. On the other hand, dietary fibers also affected the metabolic pattern of tannic acid, which was related to the dietary fiber type. In future, we will elucidate the interplay between dietary fibers and polyphenols and explore targeted combinations to modulate metabolic transformations and achieve precision nutrition. In addition, it is also indispensable to further study the synergistic effect of polyphenols and dietary fibers on human health through *in vivo* and human clinical trials.

## Data Availability

The datasets presented in this study can be found here: https://ngdc.cncb.ac.cn/bioproject, accession number CRA033735.

## Footnotes

1
https://www.r-project.org/
